# Third dose of anti-SARS-CoV-2 inactivated vaccine for patients with RA: Focusing on immunogenicity and effects of RA drugs

**DOI:** 10.3389/fmed.2022.978272

**Published:** 2022-08-31

**Authors:** Ting Zhao, Bo Wang, Jiayan Shen, Yuanyuan Wei, Youyang Zhu, Xiaofang Tian, Guangfen Wen, Bonan Xu, Chenyang Fu, Zhaohu Xie, Yujiang Xi, Zhenmin Li, Jiangyun Peng, Yang Wu, Xiaohu Tang, Chunping Wan, Lei Pan, Wenxin Zhu, Zhaofu Li, Dongdong Qin

**Affiliations:** ^1^School of Basic Medical Sciences, Yunnan University of Chinese Medicine, Kunming, China; ^2^The First School of Clinical Medicine, Yunnan University of Chinese Medicine, Kunming, China; ^3^The Department of Educational Administration, Yunnan University of Chinese Medicine, Kunming, China; ^4^The Third Affiliated Hospital, Yunnan University of Chinese Medicine, Kunming, China; ^5^The Second School of Clinical Medicine, Yunnan University of Chinese Medicine, Kunming, China; ^6^Department of Rehabilitation, The People's Hospital of Yunxian, Yunxian, China

**Keywords:** COVID-19, inactivated SARS-CoV-2 vaccines, rheumatoid arthritis, immunogenicity, Traditional Chinese Medicine

## Abstract

**Objectives:**

To evaluate the immunogenicity of the third dose of inactivated SARS-CoV-2 vaccine in rheumatoid arthritis (RA) patients and explore the effect of RA drugs on vaccine immunogenicity.

**Methods:**

We recruited RA patients (*n* = 222) and healthy controls (HC, *n* = 177) who had been injected with a third dose of inactivated SARS-CoV-2 vaccine, and their neutralizing antibody (NAb) titer levels were assessed.

**Results:**

RA patients and HC were age- and gender-matched, and the mean interval between 3rd vaccination and sampling was comparable. The NAb titers were significantly lower in RA patients after the third immunization compared with HC. The positive rate of NAb in HC group was 90.4%, while that in RA patients was 80.18%, and the difference was significant. Furthermore, comparison of NAb titers between RA treatment subgroups and HC showed that the patients in the conventional synthetic (cs) disease-modifying anti-rheumatic drugs (DMARDs) group exhibited no significant change in NAb titers, while in those receiving the treatment of biological DMARDs (bDMARDs), Janus Kinase (JAK) inhibitors, and prednisone, the NAb titers were significantly lower. Spearman correlation analysis revealed that NAb responses to SARS-CoV-2 in HC did differ significantly according to the interval between 3rd vaccination and sampling, but this finding was not observed in RA patients. In addition, NAb titers were not significantly correlated with RA-related laboratory indicators, including RF-IgA, RF-IgG, RF-IgM, anti-CCP antibody; C-RP; ESR; NEUT% and LYMPH%.

**Conclusion:**

Serum antibody responses to the third dose of vaccine in RA patients were weaker than HC. Our study will help to evaluate the efficacy and safety of booster vaccination in RA patients and provide further guidance for adjusting vaccination strategies.

## Introduction

Severe acute respiratory syndrome coronavirus 2 (SARS-CoV-2) is a positive-sense single-stranded RNA virus that is highly contagious (1). After infected with SARS-CoV-2, patients may be accompanied by symptoms such as cough, fever, and chest discomfort, which can be life-threatening in severe cases (2). According to the latest data, 579,092,623 confirmed cases of COVID-19 have been reported worldwide. Furthermore, approximately 6.41 million people have died from COVID-19 as of August 8, 2022 (https://covid19.who.int). Variants of concern have appeared at regular intervals—alpha, beta, gamma, delta, and now omicron. The omicron variant has stronger infectivity and faster transmission speed, rapidly becoming the dominant circulating variant (3). Vaccination is the most effective way to prevent and control the COVID-19 epidemic (4). It can improve the body's immunity, and currently, there are 6 different vaccines listed on the WHO Emergency Use List (EUL), namely the Pfizer/BioNTech Comirnaty vaccine, the AstraZeneca vaccine (AZD1222), the Janssen vaccine (Ad26.COV 2.S), the Moderna COVID-19 vaccine (mRNA-1273), the Sinopharm vaccine, and the Sinovac-CoronaVac (5). At least 12 billion COVID-19 vaccines have been vaccinated worldwide, which is crucial for developing immune defenses and reducing the severity and mortality (https://coronavirus.jhu.edu/map.html).

Inactivated vaccine is China's primary type of vaccine (6), which has proven to be safe and well-tolerated in healthy adults (7–9). Recent studies have found that a third dose (booster) of inactivated SARS-CoV-2 vaccine showed favorable safety profiles and restored potent SARS-CoV-2-specific immunity (10). Individuals who received three doses of mRNA vaccine responded rapidly and produced antibodies capable of clearing various variants, with increased numbers of memory B cells expressing more potent and broader antibodies (11, 12). Although many vaccinated or convalescent individuals were still infected with the omicron variant of SARS-CoV-2, three doses of inactivated vaccine can significantly reduce COVID-19 disease severity. Our previous study found that there was a significant difference in NAb levels between rheumatoid arthritis (RA) patients and healthy controls (HC) who both had received two injections of inactivated vaccine (13). However, it is still unclear whether there is a difference between RA patients and HC in antibody responses to SARS-CoV-2 induced by the third dose of inactivated vaccine.

To our knowledge, this is the first study in China focusing on the efficacy and safety of three vaccine boosters in RA patients as well as exploring the effect of RA drugs on vaccine immunogenicity. Here, we reported the immunogenicity of patients with RA to vaccination with the third dose of inactivated SARS-CoV-2 vaccine, and the correlation between RA-related indices and COVID-19 antibodies, and the effect of different drugs on the immunogenicity were also investigated.

## Methods

### Study design

**Figure 1:**
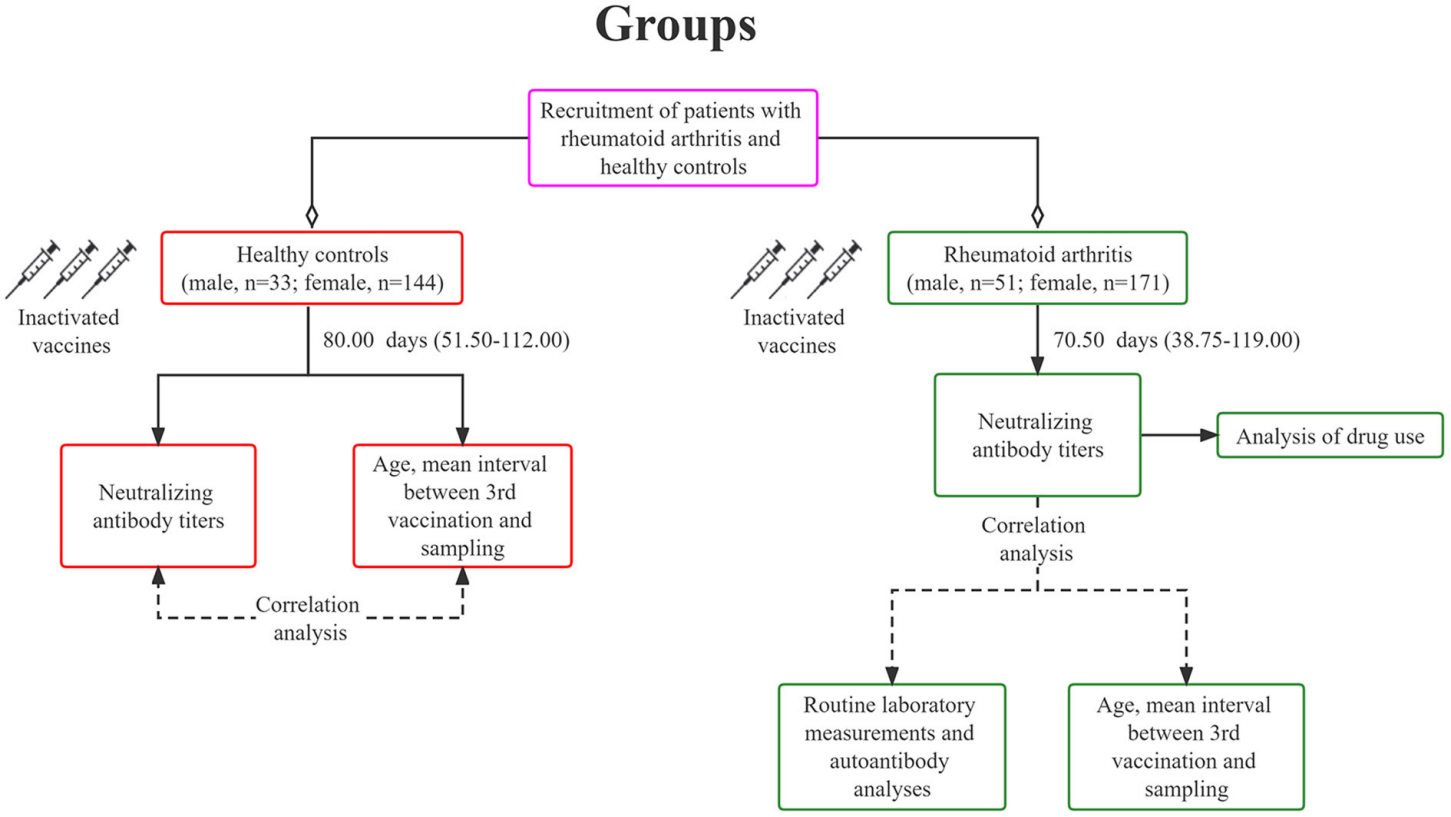
A standard flow diagram for this study.

We conducted an open-label trial in Yunnan Provincial Hospital of TCM (Yunnan, China). All participants signed written informed consent. The patient met the 1987 American College of Rheumatology (ACR) diagnostic criteria for RA. Patients with a history of COVID-19 exposure or a positive SARS-CoV-2 PCR test were excluded, and those with other serious diseases, such as severe cardiovascular and cerebrovascular diseases were also excluded. Subsequently, we invited subjects without RA or immunosuppressive therapy as the HC. All subjects were ≥18 years old and received three doses of inactivated COVID-19 vaccine, CoronaVac (3 μg/0.5mL, Sinovac Life Sciences, Beijing, China). RA patients and HC received a third dose of the vaccine primarily between December 2021 and May 2022. Blood samples were collected around January and June 2022. A total sample of 222 RA patients and 177 HC were recruited (Figure 1). Age, sex, and the interval between the third vaccination and sampling were matched between the two groups (Table 1). This study was approved by Medical Ethics Committee of Yunnan Provincial Hospital of Traditional Chinese Medicine (IRB-AF-027-2022/01-02), and has been registered on www.ClinicalTrials.gov (NCT05191368).

**Table 1:** Baseline characteristics of and RA patients and HC.

	**RA patients** **(*n* = 222)**	**HC** **(*n* = 177)**	***p*****–** **value**
Age, yrs, median (IQR)	53.23 ± 11.56	54.11 ± 12.98	0.47
Female sex, *n (%)*	171 (77.00)	144 (81.36)	0.29
Mean interval between 3rd vaccination and sampling, days, median (IQR)	70.50 (38.75–119.00)	80 (51.50–112.00)	0.27
**RA disease characteristics**			
AKA positivity, *n (%)*	138 (67.98)		
RA disease duration, yrs, median (IQR)	4.00 (1.00–10.00)		
RF–IgA, U/ml, median (IQR)	28.51 (4.72–128.42)		
RF–IgG, AU/ml, median (IQR)	112.00 (38.50–286.00)		
RF–IgM, AU/ml, median (IQR)	186.00 (47.40–829.50)		
Anti–CCP antibody, U/ml, median (IQR)	184.50 (18.90–571.50)		
ESR, mm/h, mean (SD)	29.50 (16.00–50.00)		
C–RP, mg/l, median (IQR)	7.41 (0.97–21.54)		
NEUT%, mean (SD)	64.10 (56.03–71.70)		
LYMPH%, median (IQR)	26.30 (19.13–32.80)		
**DMARD therapy**			
csDMARDs (Monotherapy), *n (%)*	76 (34.23)		
bDMARDs, *n (%)*	23 (10.36)		
JAK inhibitors, *n (%)*	24 (10.81)		
Prednisone, *n (%)*	46 (20.72)		
Immune modulating drugs (Monotherapy), *n (%)*	65 (29.27%)		
Immune modulating drugs combined with traditional Chinese medicine, *n (%)*	104 (46.85)		
**Negative symptoms after three vaccine boosters**			
Generalized weakness/fatigue	6 (2.70)	9 (5.08)	0.22
Headache	5 (2.25)	3(1.70)	0.97
Dizziness	9 (4.05)	7 (3.95)	0.96
Muscle pain/myalgia	12 (5.41)	9 (5.08)	0.89
Joint pain	5 (2.25)	0 (0)	0.12

AKA, Anti–keratin antibody; RF, Rheumatoid factor; Anti–CCP antibody, Anti–cyclic citrullinated peptide antibody; ESR, Blood sedimentation; C–RP, C–reactive protein; NEUT, Neutrophils; LYMPH, Lymphocytes; DMARDs, Disease modifying antirheumatic drugs; cs, Conventional synthetic; b, Biologic; JAK, Janus kinase.

### Anti-SARS-CoV-2 NAb measurement

Serum samples were collected, and the anti-SARS-CoV-2 NAb quantitative detection kit (Spike RBD) (RAS-N044, ACROBiosystems, Beijing, China) was used to measure the NAbs titers. The NAbs against the SARS-CoV-2 Spike RBD mutation were detected in samples by competitive ELISA. The microplates in the kit are pre-coated with human ACE2 protein. To start the experiment, add the samples and calibrators to the wells, followed by the HRP-SARS-CoV-2 Spike RBD. After incubation, the wells are washed, and the substrate solution is added to the wells. The reaction was terminated by adding a stop solution. The NAbs in the sample will compete with ACE2 for HRP-SARS-CoV-2 Spike RBD binding. The intensity of the detected signal decreased proportionally to the concentration of neutralizing antibodies against SARS-CoV-2. Detection range: 10.18 IU/mL-135.28 IU/mL. The cut-off of this kit is 10.18. The NAb titer ≥ 10.18 IU/mL is positive. Otherwise, it is negative. A Variskan flash automatic microplate reader (Thermo Scientific, USA) was used to measure absorbance at 450 nm.

### Routine clinical testing

Blood samples were collected from RA patients as part of routine laboratory measurements. NEUT% (percent of neutrophils) and LYMPH% (percent of lymphocytes) were calculated by computational methods. Rheumatoid factor (RF)-IgA was detected by ELISA (14). RF-IgG, RF-IgM, and anti-CCP antibodies were measured by chemiluminescence (15). Erythrocyte sedimentation rate (ESR) was detected by using the Westergren method. C-reactive protein (CRP) was detected by immunoturbidimetry (16). Anti-keratin antibody (AKA) was detected by indirect immunofluorescence assay (14).

### Statistical analysis

All acquired data were statistically analyzed with SPSS 26.0. If the data followed the normal distribution, they were presented as mean ± SD (standard deviation), and two independent sample *t*-tests were used. Non-normally distributed data were presented as median (IQR), and analyzed using the Mann-Whitney U test (two groups) or William Kruskal (three groups and more). Categorical variables were presented as numbers (percentage) and analyzed using χ2 tests. Spearman rank correlation coefficient was used to measure the degree of associations between laboratory parameters and NAb titers *p* < 0.05 was considered statistically significant.

## Results

### Participant's characteristics

As illustrated in Table 1, the ages of vaccinated RA patients and HC were 53.23 ± 11.56 years and 54.11 ± 12.98 years, respectively, with no significant difference (*p* = 0.47). Among the RA patients, there were 51 males (22.97%) and 171 females (77.03%). While, in HC, 33 were males (18.64%) and 144 were females (81.36%). No significant difference was evident (*p* = 0.292). The interval between the third vaccination and serum sampling in RA patients was comparable to that in HC (RA vs. HC: 70.50 (38.75–119.00) days vs. 80 (51.50–112.00) days, *p* = 0.27). All RA patients received continuous treatment with conventional synthetic (cs), biological (b), or targeted synthetic (ts) disease-modifying anti-rheumatic drugs (DMARDs) along with Janus kinase (JAK) inhibitors, prednisone, or traditional Chinese medicine (TCM). Seventy-six (34.23%) patients received therapy of csDMARDs alone and 23 (10.36%) patients received treatment of bDMARDs. Twenty-four (10.81%) patients were taking JAK inhibitors, and 46 (20.72%) patients were taking prednisone. Further analysis showed that 65 (29.27%) patients received monotherapy of immunomodulatory agents, including 28 (12.61%) csDMARDs (monotherapy), 6 (2.70%) bDMARDs, 10 (5.50%) JAK inhibitors, and 21 (9.46%) prednisone. 104 patients (46.85%) received combined treatments of immune modulating drugs and TCM, including 48 (21.62%) csDMARDs (monotherapy), 17 (7.66%) bDMARDs, 14 (6.30%) JAK inhibitors, and 25 (11.26%) prednisone. TCM included Tripterygium Glycosides Tablets, Biqi capsules and Juan Bi Granules.

### Vaccine safety

The third dose of inactivated SARS-CoV-2 vaccine was safe in RA patients. Side effects and adverse reactions in the RA and HC were comparable, and no serious adverse events were reported. RA patients and healthy controls reported negative symptoms after three vaccine boosters, such as generalized weakness/fatigue, headache, dizziness, and muscle pain/myalgia. There were no significant differences in adverse reactions between the two groups (Table 1, all *p*-values > 0.05). It is worth noting that five patients (2.25%) experienced the aggravation of joint pain after vaccination, but this may not be caused by the vaccination, and the specific reasons needed to be further explored (Table 1).

### Neutralizing antibody (NAb) titers

**Figure 2:**
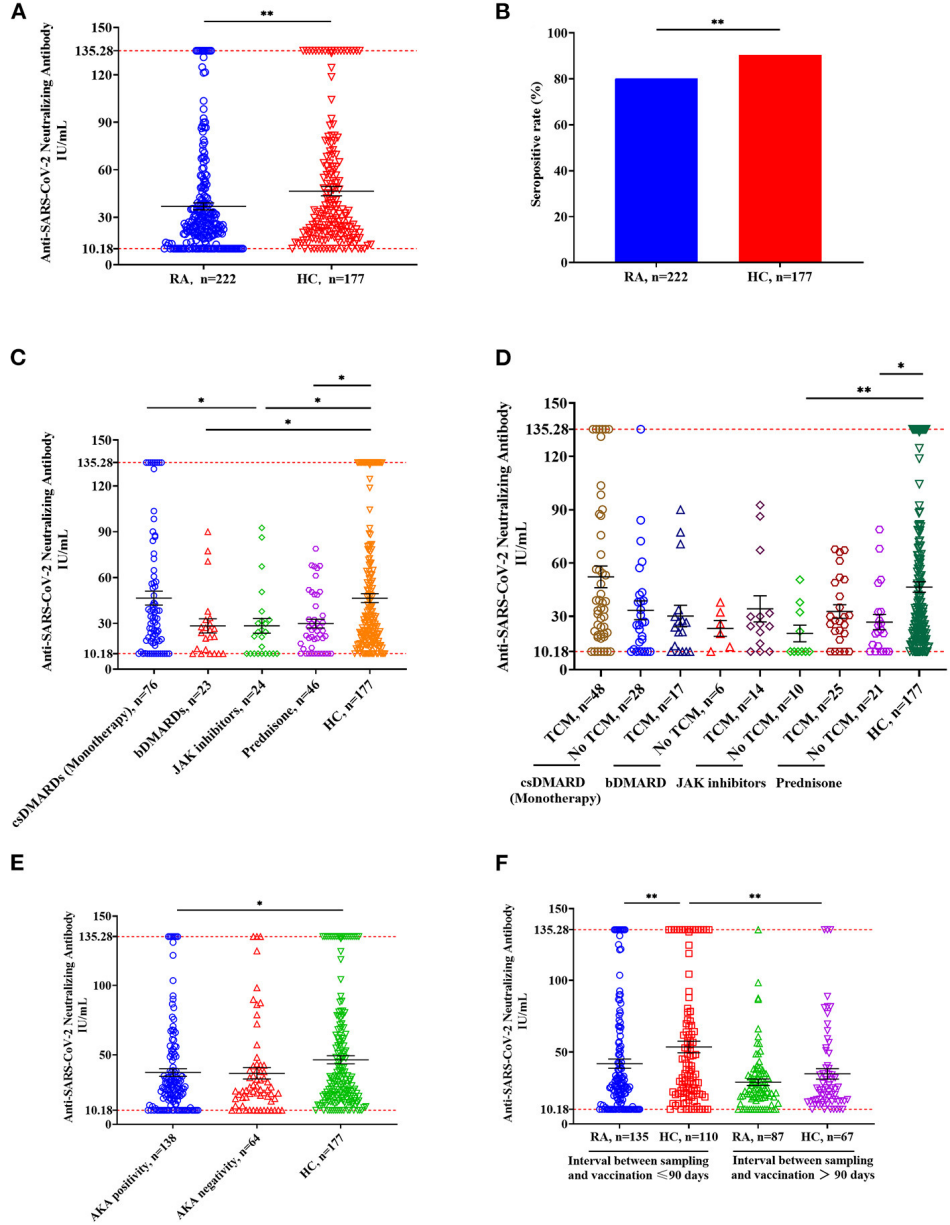
Anti-SARS-CoV-2 NAbs responses from RA patients and HC. **(A)** NAb titers in RA patients (*n* = 222) and HC (*n* = 177). Symbols show individual values, and the red line shows the maximum value, and the black horizontal bar shows the median. Statistical analysis was performed using the Mann-Whitney U test. **(B)** Seropositive rates in RA patients and HC. Statistical analysis was performed using the χ^2^ test. **(C)** Effects of immunomodulatory drugs on NAb titers. RA patients were divided into four groups based on the immunomodulatory drug they were using, including csDMARD (*n* = 76), bsDMAD (*n* = 24), JAK inhibitors (*n* = 24) and prednisone (*n* = 46). **(D)** Effects of TCM on NAb titers. Based on whether they were taking TCM, each RA group mentioned in Figure 2C was further divided into two groups including TCM and no TCM. **(E)** NAb titers in RA patients with anti-keratin antibody positive or negative compared with HC. **(F)** Comparison of NAb titers in groups having different interval time. ^*^p < 0.05; ^**^p < 0.01.

NAb titers were significantly lower in RA patients who received three doses of vaccine compared to the HC (Figure 2A, RA: median 25.20, IQR 15.27–41.47; HC: median 30.21, IQR 18.60–62.27; *p* = 0.008). NAb positivity was 80.18% in RA patients and 90.4% in HC (Figure 2B, *p* = 0.005). Compared with the HC, RA patients treated with bDMARDs, JAK inhibitors, and prednisone had significantly lower NAb titers (Figure 2C, all *p-*values < 0.05), but no significant decrease was found in RA patients treated with csDMARDs (Figure 2C, *p* = 0.996). Compared with the RA patients treated with csDMARDs, NAb titers were significantly lower in patients taking JAK inhibitors (Figure 2C, *p* = 0.027). Compared with the HC, no significant change in NAb titers was observed in RA patients receiving a combination treatment of JAK inhibitors/prednisone and TCM, while the RA patients receiving monotherapy of JAK inhibitors and prednisone had lower NAb titers than HC (Figure 2D, JAK inhibitors: *p* = 0.006; prednisone: *p* = 0.015). In addition, the anti-keratin antibody (AKA)-positive patients had significantly lower NAb titers than HC (Figure 2E, *p* = 0.039). NAb titers were significantly lower in RA patients than those in HC when the sampling time was within 90 days of vaccination (Figure 2F, *p* = 0.007). When the interval time between sampling and vaccination was more than 90 days, there was no significant difference in NAb titers between the two groups (Figure 2F, *p* = 0.498). Furthermore, there was a significant difference in NAb titers between HC ≤ 90 days and HC > 90 days (Figure 2F, *p* = 0.003), but this difference was not observed in RA patients.

### NAb titers in relation to levels of laboratory indicators

**Figure 3:**
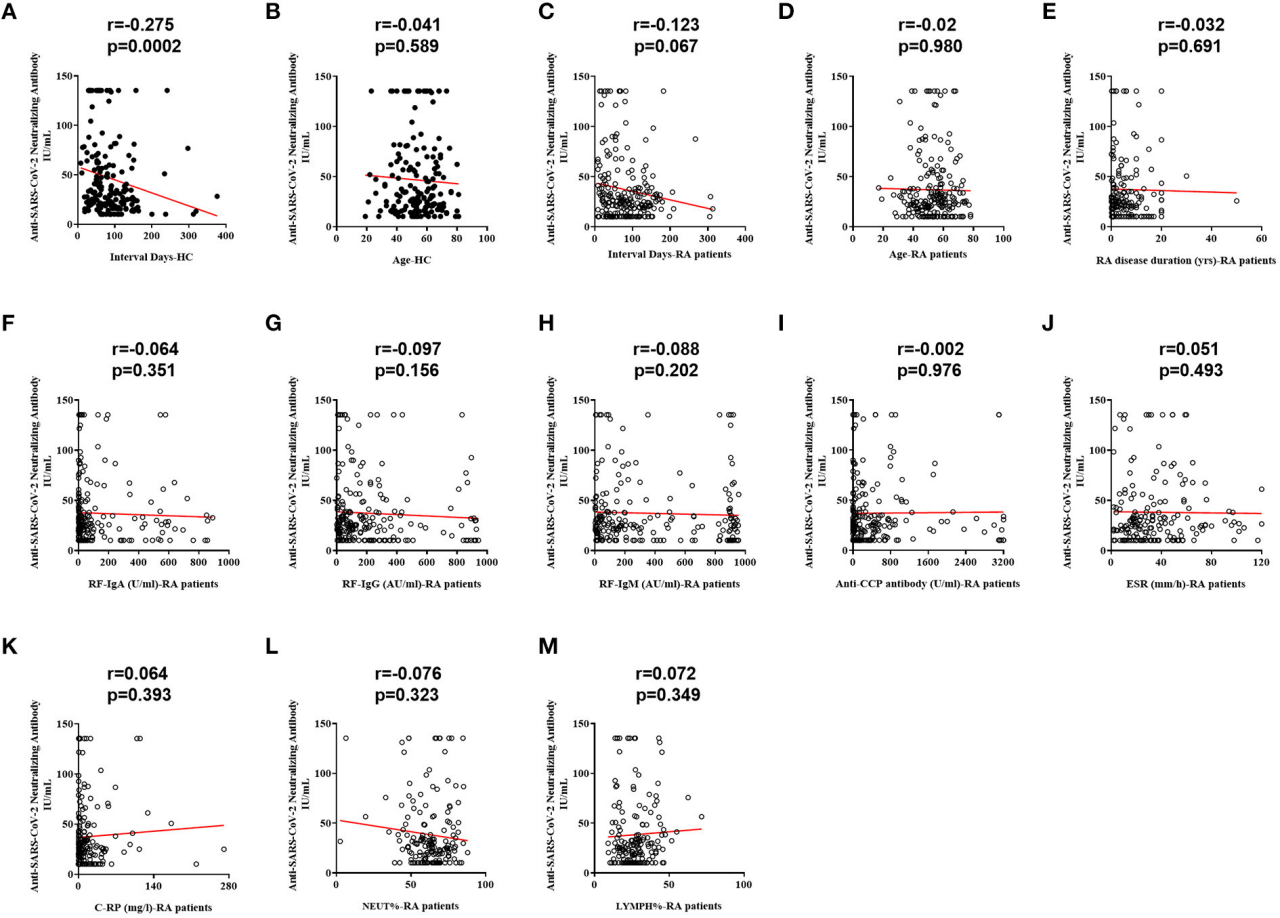
Correlations between RA-related indicators and NAb titers. **(A)** Correlation between the interval time and NAb titers in HC. **(B)** Correlation between age and NAb titers in HC. **(C)** Correlation between the interval time and NAb titers. **(D)** Correlation between age and NAb titers in RA patients. **(E)** Correlation between disease duration and NAb titers in RA patients. **(F)** Correlation between RF-IgA and NAb titers in RA patients. **(G)** Correlation between RF-IgG and NAb titers in RA patients. **(H)** Correlation between RF-IgM and NAb titers in RA patients. **(I)** Correlation between anti-CCP antibody and NAb titers in RA patients. **(J)** Correlation between ESR and NAb titers in RA patients. **(K)** Correlation between C-RP and NAb titers in RA patients. **(L)** Correlation between NEUT% and NAb titers in RA patients. **(M)** Correlation between LYMPH% and NAb titers in RA patients.

Spearman correlation analysis revealed that NAb responses to SARS-CoV-2 in HC did differ according to intervals between the third vaccination and sampling (Figure 3A, r = −0.275, *p* = 0.0002), but this correlation was not significant in RA patients (Figure 3C, r = −0.123, *p* = 0.067). No significant relations to age were found in both HC (Figure 3B, r = −0.041, *p* = 0.589) and RA groups (Figure 3D, r = −0.02, *p* = 0.980). There was also no significant correlation between disease duration and NAb titers (Figure 3E, r = −0.032, *p* = 0.691). Further results showed that NAb titers were not related to levels of laboratory indicators, including RF (Figure 3F, IgA: r = −0.064, *p* = 0.351; Figure 3G, IgG: r = −0.097, *p* = 0.156; and Figure 3H, IgM: r = −0.088, *p* = 0.202), anti-cyclic citrullinated peptide antibody (Figure 3I, anti-CCP antibody: r = −0.002, *p* = 0.976), erythrocyte sedimentation rate (Figure 3J, ESR: r = 0.051, *p* = 0.493) and C-reactive protein (Figure 3K, C-RP: r = 0.064, *p* = 0.393), as well as NEUT% (Figure 3L, r = −0.076, *p* = 0.323) and LYMPH% (Figure 3M, r = 0.072, *p* = 0.349).

## Discussion

NAb titer levels are positively correlated with vaccine protection, and ongoing disease surveillance studies can be conducted to assess vaccine protection's durability better. Studies have found that three doses of mRNA vaccine increased serum antibody responses to multiple SARS-CoV-2 variants (17, 18). A booster with SARS-CoV-2 vaccines can increase NAb levels and prevent the infection of omicron or future variants (19). It has been demonstrated that immunosuppression is associated with diminished NAb positivity (20). We found that patients with RA who received three injections of inactivated SARS-CoV-2 vaccine had significantly lower NAb titers than HC. Furthermore, RA patients had significantly lower rates of NAb positivity than HC. Therefore, the vaccine's protective efficacy in RA patients may be weaker than that of HC. The detailed analysis provided new evidence that, in many different combinations, the immune response was reduced overall. After three vaccinations, csDMARD titers were not significantly decreased, whereas patients taking tsDMARD had significantly lower titers than HC, consistent with a previous study (21). In addition, bsDMARD and prednisone can also significantly reduce the level of NAb titers. The underlying mechanism of these drugs affecting NAbs needs further experimental exploration.

Since the outbreak of the COVID-19, many countries and various localities have successively introduced a series of treatment plans in which TCM has been incorporated into. Clinical and scientific research data have verified TCM's safety and effectiveness (22–24). In this study, NAb titers showed an overall upward trend in patients treated with a combination of TCM and immune-modulating drugs, whereas NAb titers were significantly lower in patients treated with JAK inhibitors or prednisone alone than HC. This suggested that TCM may promote the immunogenicity of vaccines in RA patients. It is well known that vaccines composed of antigens alone can only stimulate weak immunogenicity and TCM ingredients can be used as adjuvant to enhance the immunogenicity of the antigens (25, 26). Recent studies have found that TCM polysaccharides can regulate the immune response by activating the signal pathway of natural killer cells, T/B lymphocytes, complement system, and so on (27, 28). However, the composition of TCM is relatively complex, and the underlying mechanisms of TCM in regulating immunogenicity of patients with RA need further experimental verification. In addition, AKA is associated with RA disease activity, which can be used for the early diagnosis (29). AKA positive patients were more severe than negative patients in terms of joint swelling index, joint tenderness index, rest pain, morning stiffness time, and joint damage. Our result showed that NAb titers of AKA-positive patients were significantly lower than those of HC. Therefore, AKA positivity may be an essential factor affecting NAb titers, and further research should be designed to verify it. The interval day between sampling and vaccination may be a crucial factor influencing the protective efficacy of vaccine, which is consistent with the previous research (30).

In conclusion, our study showed that serum antibody responses to the third dose of vaccine in RA patients were weaker than HC. Our study will help to evaluate the efficacy and safety of booster vaccination in RA patients and provide further guidance for adjusting vaccination strategies.

## Data availability statement

The original contributions presented in the study are included in the article/supplementary material, further inquiries can be directed to the corresponding author.

## Ethics statement

The studies involving human participants were reviewed and approved by Medical Ethics Committee of Yunnan Provincial Hospital of Traditional Chinese Medicine (IRB-AF-027-2022/01-02). The patients/participants provided their written informed consent to participate in this study.

## Author contributions

All authors listed have made a substantial, direct, and intellectual contribution to the work and approved it for publication.

## Funding

This work was supported by the National Natural Science Foundation of China (31960178, 82160923, 81960863, 82160901, and 81960870), Construction Project of National TCM Clinical Research Base (2018 No. 131), Yunnan Provincial Fund for Medical Research Center: Clinical Evaluation and Basic Research on the Treatment of Rheumatoid Arthritis and Gout by TCM (202102AA310006), Clinical Trial for the Treatment of Rheumatoid Arthritis with Warming yang and Smoothening Meridians (201507001-07, registration number: ChiCTR-INR-16010290), Clinical Cooperative Project of Chinese and Western Medicine for Major and Knotty Diseases, Yunnan Provincial Key Laboratory Construction Project Funding, Yunnan Provincial Key Laboratory of Chinese Medicine Rheumatology and Immunology, Yunnan Provincial Ten Thousands Program Famous Doctor Special, Yunnan Province Qingguo Wang Expert Workstation Construction Project (202005AF150017), Yunnan Applied Basic Research Projects- Union Foundation [2019FF002(-031)], Applied Basic Research Programs of Science and Technology Commission Foundation of Yunnan Province (2019FA007), Key Laboratory of Traditional Chinese Medicine for Prevention and Treatment of Neuropsychiatric Diseases, Yunnan Provincial Department of Education, Scientific Research Projects for High-level Talents of Yunnan University of Chinese Medicine (2019YZG01), and Young Top-Notch Talent in 10,000 Talent Program of Yunnan Province (YNWR-QNBJ-2019-235).

## Conflict of interest

The authors declare that the research was conducted in the absence of any commercial or financial relationships that could be construed as a potential conflict of interest.

## Publisher's note

All claims expressed in this article are solely those of the authors and do not necessarily represent those of their affiliated organizations, or those of the publisher, the editors and the reviewers. Any product that may be evaluated in this article, or claim that may be made by its manufacturer, is not guaranteed or endorsed by the publisher.

## References

[1] Narayanan N Nair DT. Vitamin B12 may inhibit RNA-dependent-RNA polymerase activity of nsp12 from the SARS-CoV-2 virus. IUBMB Life. (2020) 72:2112–20. 10.1002/iub.2359 32812340 PMC7461454

[2] Zhang JJ Dong X Cao YY Yuan YD Yang YB Yan YQ. Clinical characteristics of 140 patients infected with SARS-CoV-2 in Wuhan, China. Allergy. (2020) 75:1730–41. 10.1111/all.14238 32077115

[3] Karim SSA Karim QA. Omicron SARS-CoV-2 variant: a new chapter in the COVID-19 pandemic. Lancet. (2021) 398:2126–8. 10.1016/S0140-6736(21)02758-6 34871545 PMC8640673

[4] Thomas SJ Moreira ED. Jr. Kitchin N Absalon J Gurtman A Lockhart S. Safety and efficacy of the BNT162b2 mRNA Covid-19 vaccine through 6 months. N Engl J Med. (2021) 385:1761–73. 10.1056/NEJMoa2110345 34525277 PMC8461570

[5] Wang H Zhang Y Huang B Deng W Quan Y Wang W. Development of an Inactivated Vaccine Candidate, BBIBP-CorV, with Potent Protection against SARS-CoV-2. Cell. (2020) 182:713–21.e9. 10.1016/j.cell.2020.06.008 32778225 PMC7275151

[6] Han B Song Y Li C Yang W Ma Q Jiang Z. Safety, tolerability, and immunogenicity of an inactivated SARS-CoV-2 vaccine (CoronaVac) in healthy children and adolescents: a double-blind, randomised, controlled, phase 1/2 clinical trial. Lancet Infect Dis. (2021) 21:1645–53. 10.1016/S1473-3099(21)00319-4 34197764 PMC8238449

[7] Wu Z Hu Y Xu M Chen Z Yang W Jiang Z. Safety, tolerability, and immunogenicity of an inactivated SARS-CoV-2 vaccine (CoronaVac) in healthy adults aged 60 years and older: a randomised, double-blind, placebo-controlled, phase 1/2 clinical trial. Lancet Infect Dis. (2021) 21:803–12. 10.1016/S1473-3099(20)30987-7 33548194 PMC7906628

[8] Zhang Y Zeng G Pan H Li C Hu Y Chu K. Safety, tolerability, and immunogenicity of an inactivated SARS-CoV-2 vaccine in healthy adults aged 18-59 years: a randomised, double-blind, placebo-controlled, phase 1/2 clinical trial. Lancet Infect Dis. (2021) 21:181–92. 10.1016/S1473-3099(20)30843-4 33217362 PMC7832443

[9] Xia S Zhang Y Wang Y Wang H Yang Y Gao GF. Safety and immunogenicity of an inactivated SARS-CoV-2 vaccine, BBIBP-CorV: a randomised, double-blind, placebo-controlled, phase 1/2 trial. Lancet Infect Dis. (2021) 21:39–51. 10.1016/S1473-3099(20)30831-8 33069281 PMC7561304

[10] Zhang Y Yang Y Qiao N Wang X Ding L Zhu X. Early assessment of the safety and immunogenicity of a third dose (booster) of COVID-19 immunization in Chinese adults. Front Med. (2022) 16:93–101. 10.1007/s11684-021-0914-x 35122211 PMC8815383

[11] Shroff RT Chalasani P Wei R Pennington D Quirk G Schoenle MV. Immune responses to two and three doses of the BNT162b2 mRNA vaccine in adults with solid tumors. Nat Med. (2021) 27:2002–11. 10.1038/s41591-021-01542-z 34594036 PMC9004706

[12] Wang K Jia Z Bao L Wang L Cao L Chi H. Memory B cell repertoire from triple vaccinees against diverse SARS-CoV-2 variants. Nature. (2022) 603:919–25. 10.1038/s41586-022-04466-x 35090164 PMC8967717

[13] Zhao T Shen J Zhu Y Tian X Wen G Wei Y. Immunogenicity of inactivated SARS-CoV-2 vaccines in patients with rheumatoid arthritis: a case series. Front Public Health. (2022) 10:875558. 10.3389/fpubh.2022.875558 35548080 PMC9081335

[14] Agrawal S Misra R Aggarwal A. Autoantibodies in rheumatoid arthritis: association with severity of disease in established RA. Clin Rheumatol. (2007) 26:201–4. 10.1007/s10067-006-0275-5 16572283

[15] Shen R Ren X Jing R Shen X Chen J Ju S. Rheumatoid factor, anti-cyclic citrullinated peptide antibody, C-reactive protein, and erythrocyte sedimentation rate for the clinical diagnosis of rheumatoid arthritis. Lab Med. (2015) 46:226–9. 10.1309/LMZYTSO5RHIHV93T 26199263

[16] Moutachakkir M Lamrani Hanchi A Baraou A Boukhira A Chellak S. Immunoanalytical characteristics of C-reactive protein and high sensitivity C-reactive protein. Ann Biol Clin (Paris). (2017) 75:225–9. 10.1684/abc.2017.1232 28377336

[17] Wang CY Hwang KP Kuo HK Peng WJ Shen YH Kuo BS. A multitope SARS-CoV-2 vaccine provides long-lasting B cell and T cell immunity against Delta and Omicron variants. J Clin Invest. (2022) 132:e157707. 10.1172/JCI157707 35316221 PMC9106357

[18] Silva ARD. Jr. Villas-Boas LS Tozetto-Mendoza TR Honorato L Paula A Witkin SS. Generation of neutralizing antibodies against Omicron, Gamma and Delta SARS-CoV-2 variants following CoronaVac vaccination. Rev Inst Med Trop Saõ Paulo. (2022) 64:e19. 10.1590/s1678-9946202264019 35239863 PMC8901116

[19] Hu Y Sun Q A booster with SARS-CoV-2 vaccines: protection against Omicron infection. Signal Transduct Target Ther. (2022) 7:115. 10.1038/s41392-022-00973-5 35383164 PMC8980504

[20] Shinjo SK de Souza FHC Borges IBP Dos Santos AM Miossi R Misse RG. Systemic autoimmune myopathies: a prospective phase 4 controlled trial of an inactivated virus vaccine against SARS-CoV-2. Rheumatology (Oxford). (2022) 61:3351–61. 10.1093/rheumatology/keab773 34664616 PMC8574538

[21] Tran AP Tassone D Nossent J Ding NS. Antibody response to the COVID-19 ChAdOx1nCov-19 and BNT162b vaccines after temporary suspension of DMARD therapy in immune-mediated inflammatory disease (RESCUE). RMD Open. (2022) 8:e002301. 10.1136/rmdopen-2022-002301 35577478 PMC9114315

[22] Zhou S Feng J Xie Q Huang T Xu X Zhou D. Traditional Chinese medicine shenhuang granule in patients with severe/critical COVID-19: a randomized controlled multicenter trial. Phytomedicine. (2021) 89:153612. 10.1016/j.phymed.2021.153612 34126419 PMC8161732

[23] Zhang XY Lv L Zhou YL Xie LD Xu Q Zou XF. Efficacy and safety of Xiyanping injection in the treatment of COVID-19: a multicenter, prospective, open-label and randomized controlled trial. Phytother Res. (2021) 35:4401–10. 10.1002/ptr.7141 33979464 PMC8242486

[24] Fang B Zhang W Wu X Huang T Li H Zheng Y. Shenhuang granule in the treatment of severe coronavirus disease 2019 (COVID-19): study protocol for an open-label randomized controlled clinical trial. Trials. (2020) 21:568. 10.1186/s13063-020-04498-6 32580752 PMC7312108

[25] Wang D Liu Y Zhao W. The adjuvant effects on vaccine and the immunomodulatory mechanisms of polysaccharides from traditional Chinese Medicine. Front Mol Biosci. (2021) 8:655570. 10.3389/fmolb.2021.655570 33869288 PMC8047473

[26] Liu Z Wang S Zhang J Wang Y Wang Y Zhang L. Gastrodin, a traditional Chinese medicine monomer compound, can be used as adjuvant to enhance the immunogenicity of melanoma vaccines. Int Immunopharmacol. (2019) 74:105699. 10.1016/j.intimp.2019.105699 31357132

[27] Bao XL Yuan HH Wang CZ Fan W Lan MB. Polysaccharides from Cymbopogon citratus with antitumor and immunomodulatory activity. Pharm Biol. (2015) 53:117–24. 10.3109/13880209.2014.911921 25255928

[28] Borazjani NJ Tabarsa M You S Rezaei M. Purification, molecular properties, structural characterization, and immunomodulatory activities of water soluble polysaccharides from Sargassum angustifolium. Int J Biol Macromol. (2018) 109:793–802. 10.1016/j.ijbiomac.2017.11.059 29133093

[29] Wang XP Cheng QY Gu MM Leng RX Fan YG Li BZ. Diagnostic accuracy of anti-keratin antibody for rheumatoid arthritis: a meta-analysis. Clin Rheumatol. (2019) 38:1841–9. 10.1007/s10067-019-04464-x 30810911

[30] Xin Q Wu Q Chen X Han B Chu K Song Y. Six-month follow-up of a booster dose of CoronaVac in two single-centre phase 2 clinical trials. Nat Commun. (2022) 13:3100. 10.1038/s41467-022-30864-w 35660738 PMC9166693

